# Role of Cardiac Magnetic Resonance in the Evaluation of Dilated Cardiomyopathy: Diagnostic Contribution and Prognostic Significance

**DOI:** 10.1155/2014/365404

**Published:** 2014-02-04

**Authors:** Marco Francone

**Affiliations:** Department of Radiological, Oncological and Pathological Sciences, Sapienza University of Rome, Viale Regina Elena, 324 00161 Rome, Italy

## Abstract

Dilated cardiomyopathy (DCM) represents the final common morphofunctional pathway of various pathological conditions in which a combination of myocyte injury and necrosis associated with tissue fibrosis results in impaired mechanical function. Recognition of the underlying aetiology of disease and accurate disease monitoring may be crucial to individually optimize therapeutic strategies and stratify patient's prognosis. In this regard, CMR has emerged as a new reference gold standard providing important information for differential diagnosis and new insight about individual risk stratification. The present review article will focus on the role of CMR in the evaluation of present condition, analysing respective strengths and limitations in the light of current literature and technological developments.

## 1. Introduction

The main hallmark of primary dilated cardiomyopathy (DCM) is the presence of a left or biventricular dilatation with severely impaired systolic function in the absence of abnormal loading conditions (i.e., hypertension, valve disease, etc.) or ischaemic heart disease sufficient to cause global systolic impairment [1–5].

Primary forms of disease are diagnosed in approximately 30–40% of the cases and include a series of genetic, acquired, or mixed conditions in which the pathological involvement is predominantly limited to the myocardium and associated with a strong genetic inheritance in idiopathic cases (*≈*30% of patients). In secondary DCM conversely, ventricular dilation occurs as the final stage of extensive myocardial damage which can be associated with an extremely heterogeneous group of systemic affections from autoimmune, cytotoxic or metabolic diseases [1, 6–13].

Recognition and differentiation of the underlying pathological substrate leading to ventricular dilatation may be crucial not only to specifically the target patient's therapy (e.g., treatment of heart failure symptoms versus revascularization versus immunosuppressive and/or antiviral) but also for better individual risk stratification because of the extremely variable prognostic implications associated with the different forms of disease [14–17].

Postischemic DCM, for example, is the consequence of postischemic ventricular remodelling leading to chronic heart failure which is usually associated with a worse prognosis [18–20] and may benefit from revascularization and/or secondary preventive pharmacotherapy with statins and aspirin; among nonischemic forms, the worst prognosis had been associated with infiltrative myocardial disease (including amyloidosis, sarcoidosis, or hemochromatosis) and toxic postchemotherapy conditions, whereas peripartum and idiopathic etiology had substantially better mid- and long-term survival as compared to those with other causes of heart failure [21–24].

For these reasons, routine diagnostic imaging workup of patients with DCM (including echocardiography, selective coronary angiography, and, when indicated, endomyocardial biopsy) has been integrated in the last few years with the use of cardiac magnetic resonance (CMR) which allows identifing and characterizing the presence and location of myocardial damage in most of the cases combining its unique tissue characterization capabilities with the assessment of biventricular regional and global function [25–35].

The present paper will review the role of CMR in the evaluation of DCM, analysing respective strengths and limitations in the light of the current literature and technological developments.

## 2. Epidemiology and Etiology of Disease 

The true incidence of DCM is unknown and certainly underestimated in the community since strict clinical diagnostic criteria are lacking and patients may remain asymptomatic with the diagnosis being established only by screening or postmortem examination in a relevant number of cases [36–43].

It has been reported that approximately 5–8 new cases per 100 000 population per year are diagnosed representing the third cause of heart failure after ischemia and valvular heart disease corresponding to a disease prevalence of 36 cases per 100 000 in adult populations [38, 44, 45].

Epidemiology of disease was also assessed in a large longitudinal prospective cohort study of 1426 children with DCM reporting an annual incidence of 0.57 cases per 100000 per year with a statistically significant prevalence in boys versus girls, blacks versus whites, and infants (<1 year) versus children [40].

The etiology is also extremely heterogeneous; 50% the of cases are interpreted as idiopathic disease and associated with inflammatory and immunological phenomena in 20 to 30% of the patients, whereas the other half includes a broad spectrum of various conditions such as myocarditis, ischemic heart disease, peripartum disease, hypertension, HIV infection, and toxic forms [46–52].

Among toxic forms, alcoholic cardiomyopathy is the most frequent etiology of secondary DCM and accounts for approximately 4% of all cardiomyopathies, with men having a significantly worse prognosis [53, 54] and left ventricular dilation being an early finding.

Recognition of alcohol as a potential cause of cardiomyopathy is crucial since abstinence can result in an improved ejection fraction in 50% of the patients medically treated for heart failure, and continued drinking can result in further deterioration of the cardiac function. The mechanism of alcohol-induced cardiomyopathy is unclear but may involve disturbances in intracellular calcium transients, mitochondrial disruption, decreased myofibrillar proteins, and myocyte apoptosis [54–56].

Cocaine and amphetamines (including 3,4-methylenedioxymethamphetamine or “ecstasy”) can also result in DCM due to the multifactorial causes including microvascular spasms and subsequent ischemia/infarction, direct myocyte toxicity, and tachycardia-induced injury [54, 56–58].

Among chemotoxic agents, doxorubicin and anthracyclines can cause DCM which is clinically suspected in presence of increased brain-type natriuretic peptide (BNP) occurring at an early stage of this condition [59–62].

Peripartum cardiomyopathy arises in the last month of pregnancy or within 5 months postpartum with 75% of the cases manifesting in the first 2 months after delivery [63–65]; risk factors include age older than 30 years, multiparity, twin pregnancy, African American descent, and a family history of peripartum cardiomyopathy [63, 65]. This form likely depends on pregnancy-related reduced suppressor T cell activity and may result in an autoimmune type of myocardial inflammation or activation of myocarditis. Recovery, usually within 6 months, occurs in 50% of the patients [66, 67].

In pediatric population, the majority of the cases (66%) have also idiopathic forms of disease, whereas the most common known causes are myocarditis (46%) and neuromuscular disease (26%) with 1- and 5-year rates of death or transplantation of 31% and 46%, respectively [40, 68, 69].

It is likely expectable in the future that a large number of unrecognized idiopathic cases will be correctly reassigned to genetic or familial forms of diseases with subsequent changes in the epidemiology of disease [1, 36].

## 3. Clinical Features and Pathophysiology

Dilated cardiomyopathy represents the final common morphofunctional outcome of various biologic insults. It is a combination of myocyte injury and necrosis associated with myocardial fibrosis, which results in impaired mechanical function [14, 70, 71].

With myocyte failure and cytoskeletal uncoupling, the chambers become dilated and, according to Laplace's law, parietal stress increases inducing further mechanical disadvantage due to the increased oxygen demand and subsequent worsening of the ventricular systolic performances [72].

Thus, myocardial dysfunction can cause a vicious cycle leading to more myocardial dysfunction in a process called adverse ventricular remodelling [73–75].

Clinical manifestations are obviously more often related to signs and symptoms of congestive heart failure such as dyspnoea and effort-related fatigue [6, 76].

Major arrhythmias leading to syncope, embolic events, and even sudden cardiac death may occur at any stage of the disease although strongly dependent on the extent of replacement fibrosis and ventricular dysfunction [1, 14].

Poor contractile function and stasis can also lead to the formation of mural thrombi with symptoms related to distal embolism [77].

If right ventricle (RV) is involved, signs of right heart failure (raised jugular venous pulse, hepatomegaly, ascites, and peripheral oedema) may be also present.

## 4. Histological Features of DCM

Myocardial muscle in DCM may be normal or even hypertrophied primarily as a consequence of the myocyte elongation with an in-series addition of newly formed sarcomeres causing chambers enlargement with apparently normal or only slightly increased parietal wall thickness [78–80].

Histologically, typical features of DCM include substantial hypertrophy and degeneration of myocytes related to the loss of myofibrils, varying degree of interstitial fibrosis, and presence of small cluster of lymphocytes [1, 81–83].

Tissue fibrosis may be focal or diffused and induces increased left ventricular (LV) stiffness with progressively impaired diastolic and systolic function and has direct hemodynamic consequences on the remaining contractile tissue leading to changes in the chamber geometry with decompensated eccentric hypertrophy and increased parietal wall stress [84].

Fibrotic tissue, as already mentioned above, may also be a potential trigger for lethal reentrant ventricular arrhythmias representing a strong predictor of adverse cardiac outcomes [85, 86].

## 5. CMR Acquisition Protocol and Features

Imaging features to be assessed include the evaluation and quantification of LV dilatation and systolic dysfunction and detection of possible underlying tissue abnormalities particularly myocardial fibrosis [87, 88].

Although the acquisition technique should be tailored to the specific clinical request, a standard imaging protocol in DCM should necessarily include a four-chamber horizontal long-axis, two-chamber vertical long-axis, and short-axis views using breath-hold steady-state free precession (SSFP) cine sequences with full coverage of both ventricles to provide assessment of biventricular volumes and global and regional functions (Table 1).

At present, CMR can be considered the reference technique for the quantification of ventricular volumes and functional parameters, to measure wall thickness and ventricular mass in patients with DCM [89–95].

Buser et al. have found a significantly more heterogeneous end-diastolic LV wall thickness in patients with DCM compared to a reference normal population; furthermore, the physiological gradient in systolic wall thickening between LV basal and apical segments disappears with DCM [90, 94].

Previous studies showed that RV mass is preserved in DCM patients as compared to normal subjects, whereas LV mass is significantly greater with evidence of larger trabeculae as compared to normal subjects [91].

In advanced cases, LV dysfunction may be associated with diffuse myocardial wall thinning (diastolic wall thickness < 5.5 mm) [44].

An interesting feature of balanced cine-SSFP imaging concerns the hybrid T2/T1-weighting generated by the sequence that allows the depiction of myocardial enhancement when acquired after contrast administration due to the T1-weighted shortening induced by gadolinium (Gd) administration [96–98].

T2-weighted short-tau inversion recovery (T2w-STIR) imaging using an ECG-gated triple inversion recovery (IR) technique is also recommended to depict tissue edema when an overlapping active inflammatory process is suspected such as in acute or chronic myocarditis, sarcoidosis, Takotsubo syndrome, or acute myocardial infarction [27, 99–103].

A standard acquisition protocol should also include late enhancement imaging with T1 weighted inversion recovery images acquired 10–20 minutes after contrast administration (also called late gadolinium enhancement technique or LGE), usually obtained using a segmented 2D or 3D inversion recovery gradient-echo breath-hold approach, with inversion time optimized to null myocardial signal intensity.

It is also recommended to ensure matching section position and same slice thickness between IR-CE and cine-SSFP imaging in order to obtain direct comparison between regional wall motion abnormalities and LGE findings [104].

Use of IR-CE imaging may be helpful to characterize the myocardium and to differentiate DCM patients from LV dysfunction related to CAD [87].

LGE has been described as being present in patients with DCM in 12–35% of the cases, the most common pattern being characterized by a midwall linear distribution likely representing the intramural layer of septal fibrosis which has been observed in pathologic samples [44, 86, 87, 105].

**Figure 1:**
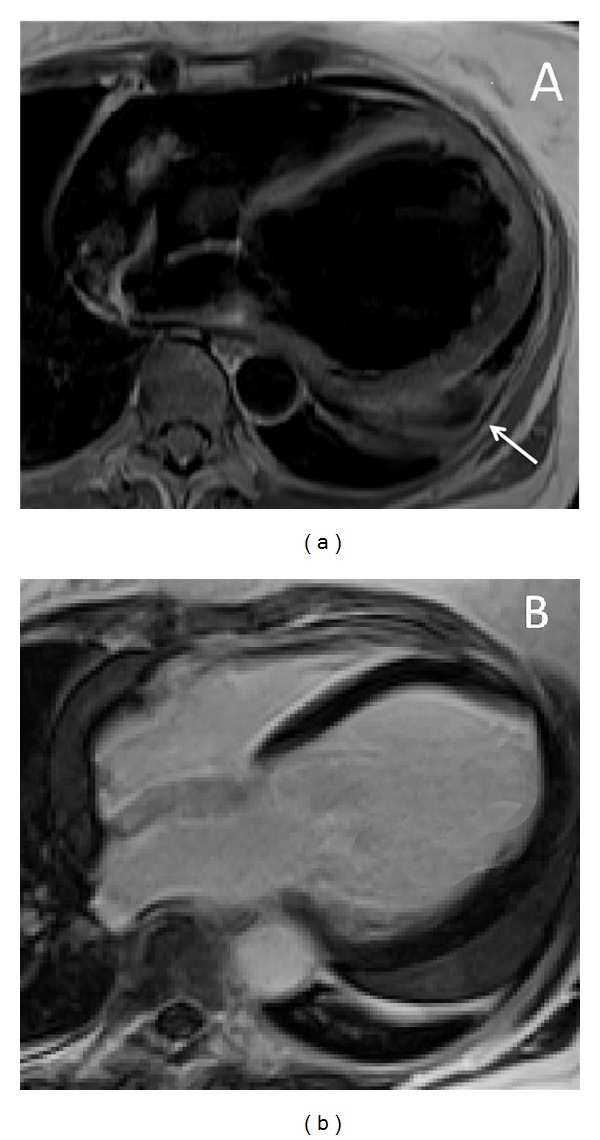
Idiopathic dilated cardiomiopathy in a 27-year-old asymptomatic patient occasionally identified during a routinary clinical screening. T1-weighted turbo spin echo 4-chamber image (a) shows a hugely dilated left ventricular chamber (end-diastolic volume 296 mL; ejection fraction 26%). Late gadolinium enhancement image acquired on the same orientation plane (b) shows absence of pathological enhancement within both ventricular chambers. There is also concomitant significant amount of pericardial effusion (arrow) located within the midbasal left ventricular lateral free wall which was initially interpreted as expression of a post pericardiomyocarditis form.

**Figure 2:**
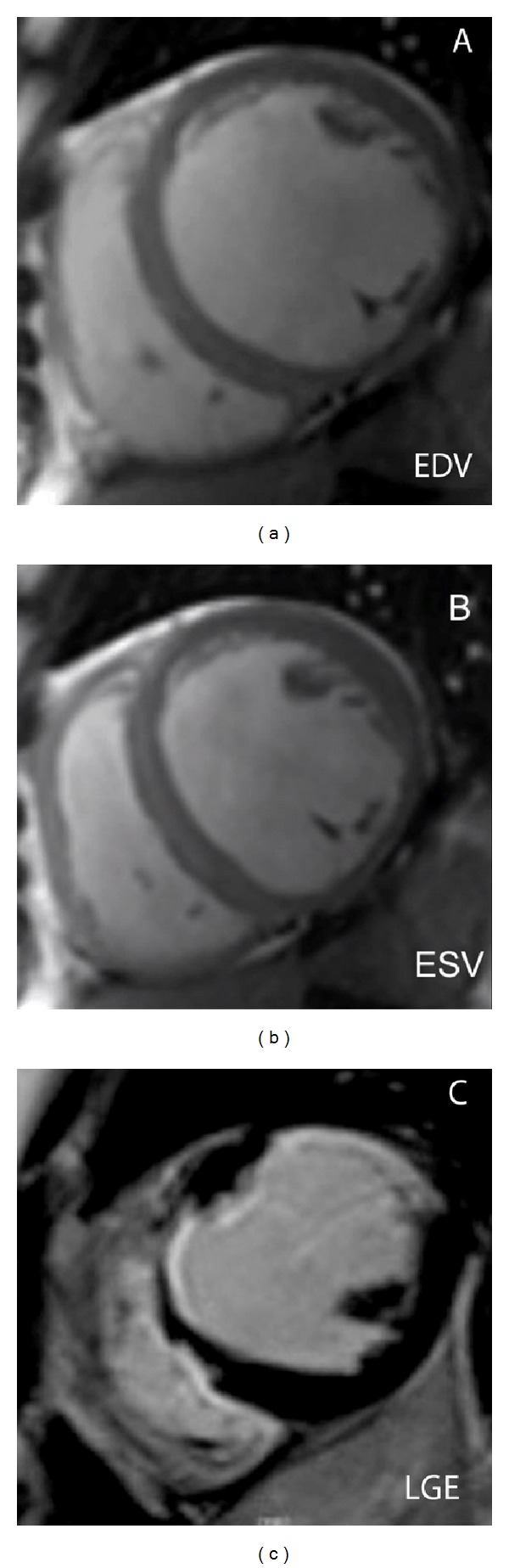
Postischemic dilated cardiomyopathy in a 69-year-old patient with severe dyspnea and symptoms of systemic heart failure. Cine-SSFP short-axis images displayed on end-diastolic (a) and end-systolic (b) phases show a markedly dilated left ventricular chamber (EDV: 287 mL) with severely compromised ejection fraction (11%). LGE image (c) depicts extensive ischemic scarring within the anterior and antero-septal and lateral walls of the left ventricle with typical subendocardial distribution of myocardial enhancement. Minimal right ventricular involvement is also present and observed as a linear hyperintense rim at the level of the right interventricular septum. Selective coronary angiography confirmed the presence of a severe 3-vessel disease which was only treated with medical therapy.

**Figure 3:**
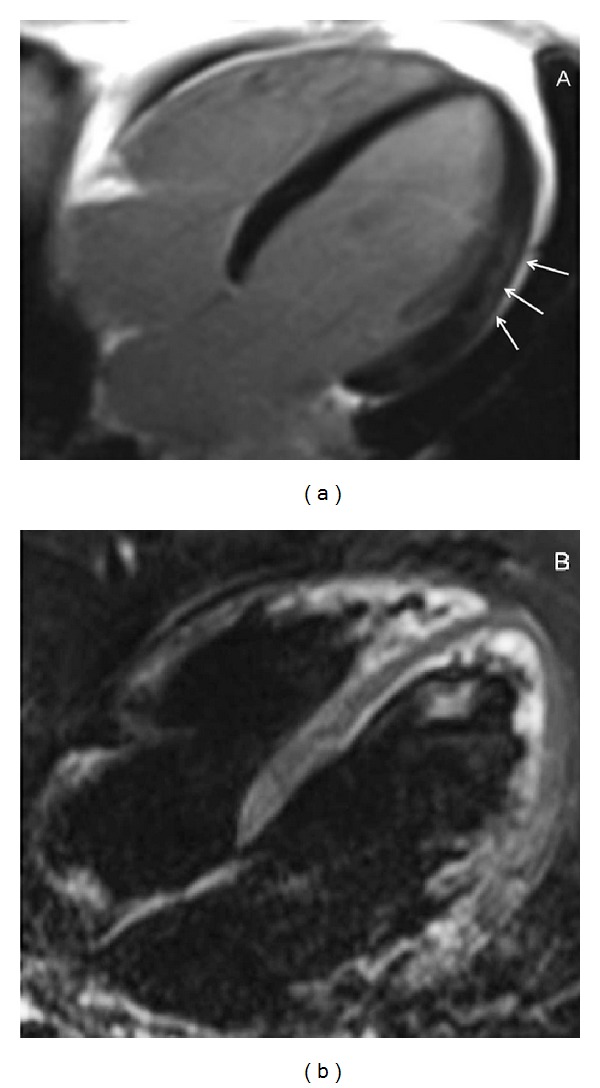
A specific midwall late enhancement stria in a 71-year-old patient with chronic dilated CMP. LGE image (a) acquired in a 4-chamber plane shows a linear post-Gd hyperintensity located within the midlateral wall of the left ventricle without corresponding to T2-STIR (b) signal abnormality. The present finding likely represents the expression of a postmyocarditic form of disease (inconfirmed in this patient).

In a previous paper, McCrohon et al. found three different LE patterns in patients with DCM: (a) no enhancement (59%; Figure 1), (b) subendocardial or transmural enhancement indistinguishable from patients with previous infarction (Figure 2), and (c) patchy or longitudinal striae of midwall enhancement clearly different from the distribution in patients with CAD (28%) (Figure 3) [87].

Transmural or subendocardial LE pattern in DCM strongly suggests the presence of a previous myocardial infarction even in the absence of remarkable coronary angiographic abnormalities and the likely explanation of this appearance may be related to spontaneous coronary recanalization after an occlusive event or a distal embolization from minimally stenotic or unstable plaques (Figure 2).

The midwall LGE pattern, rather than being expression of focal replacement fibrosis, may also represent the morphological correlate or the exitus of an inflammatory chronic process. De Cobelli et al. found LE in 70% of patients with chronic heart failure and histologically proven chronic myocarditis and midwall LE was the most frequent pattern of distribution observed in their series suggesting that IR-CE CMR may noninvasively identify areas of myocardial damage in patients presenting with chronic heart failure and no evidence of CAD, as an expression of a myocardial inflammatory process [106] (Figure 3).

Myocardial T1-mapping techniques have also been recently proposed for the depiction of diffuse myocardial fibrosis, undetectable with conventional IR-CE CMR techniques [107]. The basic principle relies on the shortening of T1-relaxation time of myocardial tissue which directly correlated with the amount of interstitial fibrosis with collagenous replacement [31].

Fast gradient-echo sequences using multiple increasing inversion times (e.g., 50–1,000 ms) before and after contrast-medium administration at the blood/myocardium equilibrium phase are performed for this purpose [108, 109].

More recently, the use of MR spectroscopy (MRS) has been proposed in the diagnostic workup of DCM [110]. The technique allows an *in vivo* noninvasive evaluation of myocardial metabolism without the need for contrast agents or radionuclides [111–116]. In CMR, hydrogen spectroscopy may be useful for assessing myocardial cellular triglyceride levels, whereas phosphorus has been used to measure myocardial energetics [117].

In a previous study, Neubauer et al. assessed 39 patients with phosphorus-31 (31P) myocardial spectroscopy and found a significantly reduced cardiovascular mortality for patients with PCr/ATP > 1.6 showing that this ratio offers significantly independent prognostic information on cardiovascular mortality [118, 119].

More recently, Dr. Beer et al. assessed the beneficial effect of the training using 31P MRS in a study population of 22 subjects and found a significant improvement in LV function without adverse effects in the metabolism further supporting the use of physical exercise as an adjunct therapy in DCM [115, 120, 121].

## 6. Clinical Indications 

CMR has evolved in the last years from an effective research tool into a clinically proven, safe, and comprehensive modality with a large spectrum of applications.

According to current guidelines and recently redefined appropriateness criteria, the use of the exam is indicated to identify the etiology of cardiac dysfunction in patients presenting with heart failure when diagnosis is unclear with conventional tools or in the evaluation of dilated cardiomyopathy in the setting of normal coronary arteries [122–127].

Current clinical indications to the exam in DCM can be schematically summarized as follows:

1. differential diagnosis between ischaemic and non-ischaemic forms;
2. preimplantation of cardiac resynchronization therapy (CRT);
3. detection of intracavitary thrombi;
4. evaluation of global biventricular function (pre-treatment and follow-up);
5. differential diagnosis in nonischaemic forms;
6. prognostic stratification which is still regarded as an investigational field of interest in this clinical setting (see the next paragraph).

### 6.1. Differential Diagnosis between Ischaemic and Nonischaemic Forms

Coronary MR angiography (MRA) for the evaluation of CAD was proposed in several publications mostly during the late 90s representing a potentially ideal technique for this purpose due to its complete noninvasiveness (no ionizing radiation and no need to administer IV contrast agents) [128–135].

Although various MRA sequences have been proposed, the best technique is currently based on a free-breathing, navigator-gated, 3D segmented GRE sequence; more recently a whole-heart SSFP CMR approach which utilizes an inferior in-plane spatial resolution was implemented [33, 136–138].

Regardless of the initial promising results, current limited spatial resolution of the exam and the presence of in-plane and through-plane coronary motion during the acquisition period prevent accurate quantification of lumen narrowing and impede visualization of distal segments making the detection of coronary stenoses in middistal segments problematic and not suitable for surgical planning [139, 140].

In a previous prospective study including 189 patients assessed with both MRA and MDCT for CAD exclusion, Dacher et al. found a significantly higher sensitivity of MDCT for detection of stenoses >50% using selective coronary angiography as a reference standard (82% versus 52%, resp.) and concluded that MDCT is superior to MRI for coronary assessment [139, 141].

For these reasons, already in 2004 clinical recommendations, coronary MRA was considered a level III diagnostic technique (i.e., infrequently used because the information from other imaging techniques is usually adequate) for direct evaluation of CAD, and similar conclusions were drawn by more recently published guidelines and appropriateness criteria [123–125, 138, 142].

The strength of CMR in CAD exclusion is conversely attributable to the utilization of pharmacologically inducible ischemia tests which allow the depiction of rest-stress perfusional changes (CMR-perfusion) or induced wall motion abnormalities (WMA-CMR) unmasking hidden myocardial ischemia [143–151].

If compared to direct competitors like stress-echo and SPECT, CMR-perfusion offers higher spatial resolution, permitting direct visualization of subendocardial ischemia with stress first-pass contrast-enhanced techniques and combining perfusional findings with IR-CE sequences allowing direct identification of subtle subendocardial lesions which are usually not visible with nuclear medicine modalities [152–154].

Rest-stress perfusion MR images are usually evaluated with semiquantitative approaches, such as an upslope analysis of myocardial time-intensity curve, or with a visual assessment in which perfusion defects are depicted on stress sequences (usually after adenosine or dipyridamole IV infusion) as regionally hypointense subendocardial dark rims, not visible on corresponding rest dynamic scans [155–157].

WMA-CMR relies on utilization of IV dobutamine stimulation which activates B-receptors of the myocardium inducing increased inotropism, heart rate, and stroke-volume at the cost of increased oxygen consumption resembling physical exercise [158]. While non-ischemic myocardial tissue will show progress contractility increase during stress testing, ischemic segments will exhibit WMA related to the presence of a flow-limiting stenosis [143, 159].

The sensitivity and specificity of high-dose dobutamine stress MRI for detecting significant CAD were reported to be, respectively, 83% and 83% by Hundley et al. and 86% and 86% by Nagel et al. and represent a valid diagnostic option for the detection of CAD associated with DCM [160–165].

### 6.2. Preimplantation of Cardiac Resynchronization Therapy (CRT)

CRT is performed in patients presenting with heart failure and DCM to provide an improvement in clinical symptoms, LV performance, and the patient's outcome. The procedure is recommended in the presence of an ejection fraction (EF) equal to or less than 35% and QRS complex duration on ECG of 120 ms or greater [166, 167] and has shown to produce a major impact on overall patient's survival resulting in risk reduction in all-cause mortality of 31% at 3-year follow-up [168].

CRT devices are biventricular pacemakers with at least two leads located in the LV cavity and coronary sinus in order to, respectively, stimulate the interventricular septum and LV lateral wall and resynchronize a heart whose walls do not contract coherently, as it happens in 25–50% of heart failure cases.

Literature however reports that there is still a significant proportion of cases (more than 30%) who do not show favourable response to device implantation and the mechanisms advocated to explain those failures included the presence of tissue scar unresponsive to cardiac pacing [169, 170]. In this regard, CMR has emerged as a valuable tool allowing simultaneously evaluating ventricular dyssynchrony using cine-SSFP or tagging techniques [171, 172] and quantifing extent and location of tissue fibrosis with IR-CE techniques as explained above. An inverse relationship between tissue scar demonstrated by CMR and CRT responses was previously shown [173] and the exam has also proved to be useful for the depiction of coronary venous anatomy to guide optimal leads placement [174].

### 6.3. Detection of Intracavitary Thrombi

Thrombus formation occurs in the presence of the so-called Virchow's triad which includes a hypercoagulability status, hemodynamic changes (stasis, and turbulence), and endothelial injury/dysfunction [175] and represents a common complication of DCM, usually underestimated with transthoracic echocardiography. In addition, because of the lack of diagnostic criteria, differentiating subacute thrombi from organized thrombi on echocardiography—a distinction that is important in predicting the risk of embolic complications—is challenging.

CMR is the preferred diagnostic tool to recognize its presence which is depicted as a soft-tissue intracavitary lesion, nonenhancing on postcontrast IR-CE images and with a variable signal intensity on either T1 or T2 images [176–178].

The technique has shown an excellent sensitivity and specificity for LV thrombus detection after the infarction and is superior to both transthoracic and transoesophageal echocardiography [179].

### 6.4. Evaluation of Global Biventricular Function (Pretreatment and Follow-Up)

Evaluation of global ventricular systolic function with cine-SSFP imaging is currently regarded as the gold standard imaging technique, not affected by the geometric assumptions used in 2D echocardiography for the left ventricle (such as the area/length method).

Furthermore, the approximation in delineating endocardial border with CMR approach is considerably less than with 2D echocardiography minimizing operator dependence and intra- and inter-observer reproducibility variability [180, 181].

In DCM, LV ejection fraction is the strongest predictor of progression to heart failure, while LV volume and mass are independently correlated with mortality and morbidity; therefore accurate quantification of all these parameters is essential for adequate patient's evaluation and also to monitor progression of disease and response to different therapeutic agents. Except for the mildly dilated forms of the disease, both ventricular chambers show a moderate to severe degree of dilatation with a severely impaired ejection fraction (e.g., lower than 20%) which requires accurate volumetric quantification and geometric follow-up changes [182, 183].

Chamber enlargement is also associated with valvular insufficiency which can be assessed with phase-contrast sequences representing an accurate technique for quantifying the severity of valve regurgitation and for providing information on diastolic function [184, 185].

### 6.5. Differential Diagnosis in Nonischaemic Forms

Differentiation between various forms of nonischemic DCM is still a complex and partially investigational issue of CMR in which the unique tissue characterization capability of the exams might offer a significant diagnostic added contribution as compared to conventional diagnostic tools [186–190].

**Figure 4:**
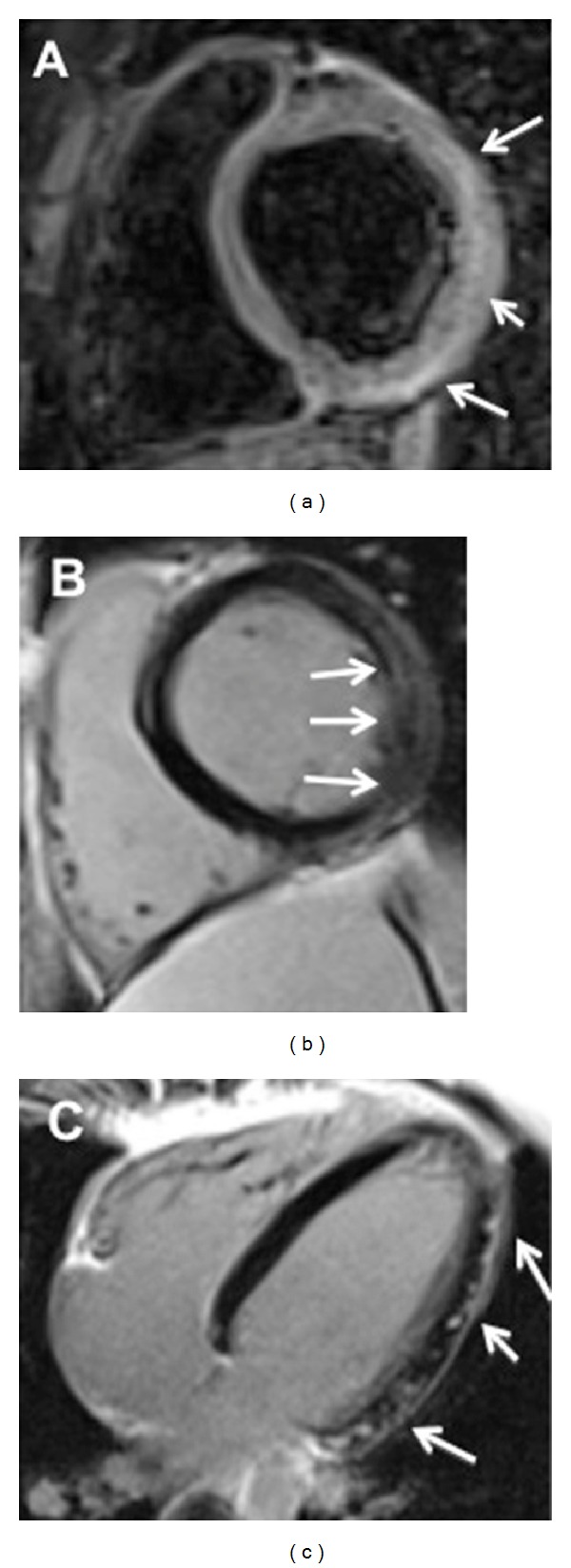
Cardiac magnetic resonance (CMR) exam performed in a 45-year-old patient with acute (5th day) myocarditis presenting a cardiomyopathic onset of disease. T2 short-tau inversion recovery image (T2-STIR) acquired on mid-ventricular short axis (a) shows subepicardial edematous imbibition of the anterior and inferolateral segment of the left ventricular myocardium (arrows) with corresponding late gadolinium enhancement with the same nonischemic pattern of distribution ((b)-(c)). Left ventricle appears moderately dilated (EDV: 203 mL); systolic function was depressed (EF: 32%).

CMR, in general, allows characterization of acute versus chronic injuries using T2w-imaging and T2-mapping techniques (Figure 4), quantification of intramyocardial irondeposition with T2^*∗*^ techniques in patients with hemochromatosis, and provides data regarding necrosis/fibrosis with LGE representing a valuable noninvasive alternative to endomyocardial biopsy [191, 192].

A pattern-based approach of LGE was previously reported in literature [193] relying on the concept that the location (subendocardial, transmural, subepicardial, or mesocardial) and pattern (focal or diffuse) of abnormal LGE allow not only differentiating between ischemic (infarct-related) and nonischemic cardiomyopathies but also ruling out differential diagnoses in cases of nonischemic forms.

Images analysis should also include evaluation of extracardiac abnormalities including the presence of pleural effusion of azygos-superior vena cava dilation which may be indirect signs of overlapped right heart failure.

Mid- or subepicardial striae of LGE represent the typical feature of postmyocarditic forms of disease in which ventricular dilatation either may occur acutely after the inflammation as the consequence of the direct damage of the cardiomyocytes by the etiologic agent causing extensive myocardial injury [194–196] or may be the consequence of a left or biventricular remodelling that induced a chronic inflammatory stimulus mediated by targeting T cells (very often from an autoimmune process) resulting in a higher and prolonged disease activity leading to ventricular dysfunction [197, 198]. Depiction of active myocardial inflammation in DCM is important as these patients may benefit and favourably respond to immunomodulatory therapy (Figure 4).

**Figure 5:**
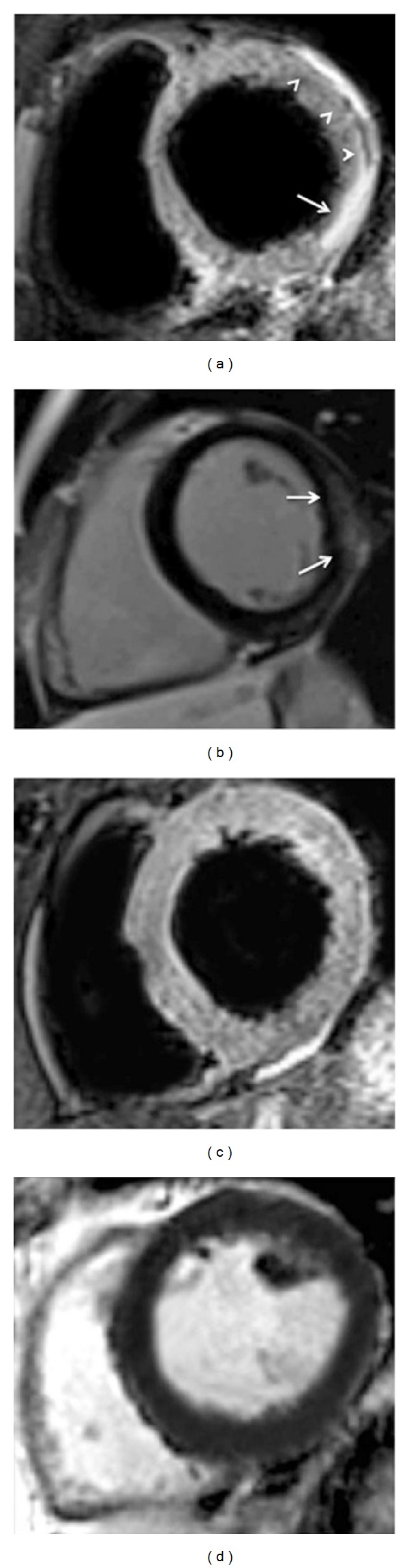
Anthracyclines induced dilated cardiomyopathy assessed with cardiac magnetic resonance at symptoms onset ((a), (b)) and eight months after drug suspension ((c), (d)). At clinical presentation, end-diastolic and end-systolic cine steady-state free-precession images show increased left ventricular volumes (EDV: 166 mL; ESV: 106 mL) with a reduced ejection fraction of 36% and a regional wall motion abnormality mostly involving apical segments. After contrast administration (b), patchy subtle areas of inhomogeneous late enhancement predominantly subepicardially distributed are depicted mostly involving the inferior and inferolateral wall of the left ventricle (arrows) and likely representing foci of replacement fibrosis related to the active inflammation associated with the drug's exposure. At follow-up ((c), (d)) after drug suspension, cine-MR shows significant recovery in global LV function (EDV 151.4 mL; ESV: 77 mL; EF: 49%) with reduced wall thickness (midseptal wall thickness from 13 mm to 10 mm). At late enhancement imaging (d), regional hyperintensity is no longer observed highly suggesting the healing of the process.

Midwall interventricular striae of LGE have also been described in patients with secondary forms of disease related to drug toxicity and alcohol abuse and again likely represent areas of replacement fibrosis, which have been reported in pathologic samples and may be related to subclinical foci of myocardial ischemia (Figure 5) [87].

One remarkable limitation to mention regarding differential diagnosis of secondary forms of the disease concerns the widespread diffusion of tissue fibrosis which might be undepictable with CMR due to the fact that LGE sequences are designed to depict focal forms of tissue fibrosis and this explains the lack of pathological enhancement reported in most of DCM cases [87].

In this regard, T1- mapping techniques were recently proposed allowing to identify of increased interstitial accumulation of gadolinium at steady state in presence of diffuse forms of disease related to expansion of extracellular space with subsequent T1-relaxation time shortening [199–202].

Tissue fibrosis depicted with T1-mapping techniques positively correlates with the degree of ventricular dilatation, contractile, and diastolic dysfunction [31, 199, 203–205].

### 6.6. Prognostic Stratification

As already mentioned above, LGE is not only a marker of differentiation between primary and secondary forms of disease but may also provide intriguing information regarding patients' risk stratification [14].

There is a large series of papers in literature reporting that the presence of mid-wall striae of tissue enhancement is predictive of inducible ventricular tachycardia allowing the stratification of patient's risk and the selection of ideal candidate for ICD [75, 86, 182, 206, 207].

Assomull et al. also correlated LGE with mortality and cardiovascular events (HR, 3.4; CI, 1.4 to 8.7) and found that it was the best predictor of sudden cardiac death (HR, 5.4) [86].

The same observation was found by Wu et al. who reported that LGE predicted adverse outcomes in patients scheduled for ICD implantation with a higher event rate (heart failure, appropriate ICD discharge, and cardiac death, 44% versus 8%; *P* = 0.001; HR, 8.2; CI, 2.2 to 30.9; *P* = 0.002) [208].

More recently, however, Hombach et al. [209] did not reproduce the same results and found that midwall enhancement was not associated with an independent prognostic impact, highlighting the prominent still investigational nature of those studies requiring further clinical validation from large prospective dedicated trials.

In this regard, a large prospective longitudinal study of 472 patients with DCM with a median follow-up of 5.3 years was recently published providing evidence that the assessment of mid-wall fibrosis with LGE-CMR imaging was independent prognostic information beyond LVEF in patients with nonischemic dilated cardiomyopathy (HR, 2.43 (95% CI, 1.50–3.92); *P* < 0.001) [14].

## 7. Conclusions

CMR has progressed in the last decade from the status of an almost fully dedicated research tool into a clinically recognized diagnostic modality with clearly established guidelines and appropriateness criteria. At present, it can be considered the real gold standard technique for the evaluation of ventricular volumes and functions, the measurement of wall thickness, and quantification of ventricular masses. Although this information can be reliably obtained with echocardiography, its added value towards competing imaging modalities relies on its unique tissue characterization potentials allowing the characterization and differentiation of various forms of DCMs with potentially relevant therapeutic and prognostic implications.

Knowledge of location, extent, and distribution of tissue fibrosis also aids potential prediction of clinical outcomes with a growing prognostic evidence as shown by recent scientific literature.

## Figures and Tables

**Table 1:** Use and significance of different CMR sequences applied for the evaluation of primary and secondary forms of DCM.

Sequence	Information provided	CMR imaging features
Cine-SSFP	Regional and global biventricular function Ventricular mass and parietal wall thickness	Dilated left or biventricular cavities Reduced ejection fraction (<40%) Parietal wall thickness normal or slightly reduced (<5.5 mm)

T2w-STIR	Myocardial free water content increase reflecting aspecific inflammatory changes	Regional hyperintense signal subendocardial involvement to rule out ischemic versus nonischemic acute disease

IR-CE or LGE	Tissue fibrosis/scar	(i) No enhancement (59%) (ii) Subendocardial or transmural enhancement indistinguishable from patients with previous infarction (iii) Patchy or longitudinal striae of mid-wall enhancement

T1-mapping	Depiction of diffuse myocardial fibrosis	Generation of T1 maps for the quantification of decay in myocardial signal intensity

MRS (hydrogen)	Assessment of myocardial cellular triglyceride	Still few data published

MRS (phosphorous)	Measurement of myocardial energetics	Reduction in PCr (~50%) and ATP (~35%), with concomitant decrease in PCr/ATP (~25%)

SSFP: steady-state free precession; T2w-STIR: T2-weighted short-tau Inversion recovery; IR-CE or LGE: Inversion recovery contrast-enhanced or late gadolinium enhancement; MRS: MR spectroscopy; PCr: phosphocreatine; ATP: adenosine-5-triphosphate (ATP).
